# Evaluation of a delirium awareness podcast for undergraduate nursing students in Northern Ireland: a pre−/post-test study

**DOI:** 10.1186/s12912-021-00543-0

**Published:** 2021-01-13

**Authors:** Gary Mitchell, Jessica Scott, Gillian Carter, Christine Brown Wilson

**Affiliations:** Queen’s University Belfast, School of Nursing and Midwifery, Medical Biology Centre, 97 Lisburn Road, Belfast, County Antrim BT9 7BL Northern Ireland

**Keywords:** Delirium, Nurse education, Nursing student, Podcast, Pedagogical research, Constructivist pedagogy, Quantitative research methods

## Abstract

**Background:**

Delirium is a common disorder affecting several people in primary, secondary, and tertiary settings. The condition is frequently under-diagnosed leading to long-lasting physical and cognitive impairment or premature death. Despite this, there has been limited research on the impact of innovative approaches to delirium education amongst undergraduate nursing students. The aim of this study was to evaluate the effect of a delirium awareness podcast on undergraduate nursing student knowledge and confidence related to the condition in Northern Ireland.

**Methods:**

The intervention was a 60-min delirium awareness podcast, available throughout May 2020, to a convenience sample of year one undergraduate nursing students (*n* = 320) completing a BSc Honours Nursing degree programme in a Northern Ireland University. The podcast focused on how nursing students could effectively recognise, manage, and prevent delirium. Participants had a period of 4 weeks to listen to the podcast and complete the pre and post questionnaires. The questionnaires were comprised of a 35-item true-false Delirium Knowledge Questionnaire (DKQ), a 3-item questionnaire about professional confidence and a 7-item questionnaire evaluating the use of podcasting as an approach to promote knowledge and confidence about delirium. Data were analysed using paired t-tests and descriptive statistics.

**Results:**

Students improved across all three core areas in the post-test questionnaire, demonstrating improvements in knowledge about symptoms of delirium (7.78% increase), causes and risk factors of delirium (13.34% increase) and management of delirium (12.81% increase). In relation to perceived confidence, students reported a 46.50% increase in confidence related to recognition of delirium, a 48.32% increase in relation to delirium management and a 50.71% increase their ability to communicate about delirium. Both questionnaires were statistically significant (*P* < 0.001). The final questionnaire illustrated that nursing students positively evaluated the use of podcast for promoting their knowledge and confidence about delirium and 96.32% of nursing students believed that the podcast met their learning needs about delirium.

**Conclusions:**

A 60-min podcast on delirium improved first year student nurse knowledge about delirium. Nursing students also expressed that this approach to delirium education was effective in their learning about the condition.

**Supplementary Information:**

The online version contains supplementary material available at 10.1186/s12912-021-00543-0.

## Background

Delirium is a common state of acute confusion which can manifest as cognitive impairment, altered consciousness levels and behavioural change usually caused by disrupted homeostasis within the body [1]. The condition can be distressing for the individual, their family members and can lead to long-term complications and can result in death [2]. The prevalence of delirium tends to rise with increasing age but it also common in very young children [1, 2]. Reporting of delirium is traditionally very poor and this indicates that awareness about the condition needs to be improved [1–4]. It is postulated that delirium affects up to 30% of hospitalised patients, nearly 80% of mechanically ventilated patients and 60% of people living in care homes [5]. Crucially, delirium is a symptom of acute illness that is often avoidable and reversible [6, 7].

Internationally, delirium is poorly recognised, and this is due to a lack of a unifying definition, a lack of education, and a lack of consistent application of clinically effective assessment tools [7–9]. In the context of the recent COVID-19 pandemic, evidence also suggests that people who contract the virus are more likely to develop delirium as a consequence [10–12]. Promoting awareness and improving healthcare professional knowledge about delirium prevention, recognition and management is therefore a key global public healthcare priority. It is demonstrated in research that the education of healthcare professional audiences is strongly correlated with improvements in knowledge about delirium and improvements in the care of people experiencing the condition [13–16].

There have been few studies that have examined the impact of delirium education on nursing students and this is surprising given their role in the assessment, planning, implementing and evaluation of patient care across primary, secondary and tertiary care settings [17–19]. In a previous paper, the authors (GM GC & CBW) described how they worked with people who had experienced delirium, specialist delirium nurses and nurse academics to co-design a face-to-face ‘delirium awareness’ programme for year one nursing students at Queen’s University Belfast [20]. This intervention showed statistically significant improvements in student nurse knowledge and confidence about delirium [20]. However, because of the recent COVID-19 pandemic and social distancing measures face-to-face delivery of this programme could not take place. As a result, the team modified the delivery of this programme to facilitate asynchronous remote learning about delirium during this period.

As a modern approach to meet the everchanging demand for nursing education, podcasts are now a widely used intervention providing learning resources [21, 22]. Podcasts can be one-off audio recording or be used to produce a series relating to a theme [21]. The evidence-base suggests that podcasts can be an engaging way to capture attention and increase engagement as often they will be able to be played at a time suitable to the individual [22–24]. This study aimed to determine the effect of a delirium awareness podcast in increasing nursing students’ knowledge of delirium and confidence in caring for individuals who experience the condition.

## Methods

### Design/setting/population

A quantitative pre-test/post-test design was used to investigate the impact of the podcast. The study was conducted with a convenience sample of year one undergraduate nursing students (*n* = 320) from Queen’s University Belfast in Northern Ireland during May 2020. All undergraduate nursing students (*n* = 320) in their first year of this nursing programme were eligible for inclusion in this study.

### Intervention

In response to the COVID-19 pandemic, all undergraduate nursing education at Queen’s University Belfast was modified for remote delivery in mid-March 2020. One topic of learning that was affected was delirium awareness. Year one nursing students were scheduled to receive a two-hour face-to-face workshop on delirium which was originally co-designed by people who had experienced delirium, expert clinicians, and academic staff [20]. The project team worked together to adapt delivery of this co-produced content to a podcast format. The podcast was developed using free software from audacity (https://www.audacityteam.org/), an external microphone and laptop computer. The time to produce this delirium awareness podcast was approximately 6 h and there were no other external costs associated with recording, editing, or publishing the podcast.

The project team worked together to design and record a 60-min podcast on delirium which was delivered by one member (GM) of the team. Due to the difficulty in supporting nursing students to engage in reflective group discussions about delirium, the podcast was embedded with two real-life stories from the experiences of the podcaster. These stories focused on a hospitalised patient living with hyperactive delirium (introduced approximately 1 min into the podcast) and a care home resident living with persistent hypoactive delirium (introduced approximately 40 min into the podcast). These narratives articulated key facts about the different types of delirium and were threaded throughout the podcast to support student understanding of the topic. In nurse education, real-life stories have a unique quality where emotion, tone and imagery illustrate the storyteller’s perspective [25]. The research team believed these meaningful stories would stimulate personal reflection and facilitate nursing students to recall important information about delirium recognition, management, and prevention.

To ensure that the previously agreed learning outcomes were met, the podcast used the previous running order from earlier co-designed education about delirium [20]. The delirium podcast threaded the two real-life stories throughout the three core elements of defining delirium, recognition of delirium and management of delirium. Table 1 provides an overview of the running order of the delirium awareness podcast.

**Table 1:** Delirium Awareness Podcast Running Order

Core Section	Themes
1. What is Delirium?	▪ A Personal Account of Delirium (Hospital-Based) ▪ Background to Delirium ▪ Prevalence of Delirium ▪ Symptoms of Delirium ▪ Types of Delirium ▪ A Personal Account of Delirium (Care Home-Based) ▪ Delirium vs. Dementia ▪ Causes of Delirium ▪ Environmental Factors
2. Recognition of Delirium	▪ NICE Recommendations on Assessment and Diagnosis of Delirium. ▪ The 4 A’s Test
3. Management of Delirium	▪ The Lived Experience of Delirium ▪ Nursing Management of Delirium ▪ Prevention of Delirium
4. Conclusion	▪ Summary and Close

The delirium awareness podcast was made available for 320 nursing students for 4 weeks throughout May and June 2020. Students were informed that listening to the delirium awareness podcast was a mandatory requirement of their year one nursing programme and they had a period of 4 weeks to do this. Students could listen to the podcast at a time convenient to them during this four-week period. The podcast was uploaded as an MP3 audio file within year one nursing student’s own Canvas Learning Management System. Canvas is the University-wide virtual learning environment for all students at Queen’s University Belfast and this system is used to support learning and teaching activities. The podcast was not available for external download. While this education formed a mandatory part of student learning, completion of the pre/post-test questionnaires did not and was voluntary.

### Data collection

Students who wished to participate in this study were informed that they needed to complete the pre and post questionnaires during the same four-week period as they received access to the podcast.

A 35-item true-false Delirium Knowledge Questionnaire (DKQ) developed by Detroyer [26], based on the work of Hare [27], was available to students to complete before and after listening to the podcast. Both pre and post questionnaires were available to students via a weblink on their University module homepage. These questionnaire weblinks were placed on the same page as the podcast and information sheets about the study.

The DKQ was developed to gauge knowledge about delirium from the perspective of healthcare professionals like nurses and doctors. This questionnaire [26] was used by the authors in their previous co-design study [20]. The DKQ was not modified and is divided into three main sections which focus on: 1) Presentation, symptoms and outcomes of delirium (*n* = 10 items), 2) Causes and risk factors of delirium (*n* = 11 items), and 3) Delirium prevention and management strategies (*n* = 14 items) [26]. The overall DKQ score received by the participant equates to the total of questions answered correctly and this ranges between 0 (lowest) and 35 (highest). Nursing students received their overall score out of 35 for each test immediately on completion. After data collection concluded, all students (including those who did not participate in this study), received a copy of the DKQ items and the correct answers to aid in the consolidation of their learning.

As in our previous delirium education study [20], the research team also administered a three-item pre−/post questionnaire about their perceived confidence of nursing patients with delirium. This short questionnaire was co-designed by people who had experienced delirium [20]. The questionnaire asked students to rate their confidence about recognition, management and communicating with family members about delirium using Likert scale items. The Likert scale items included: extremely confident, very confident, slightly confident, and not at all confident. These three items were completed after the 35-item DKQ.

The final part of data collection related to the post-test phase of the study and this focused on evaluating the effectiveness of podcasting to deliver nursing education. The authors designed a seven-item questionnaire in consultation with current university teaching evaluation guidance and the input of four current undergraduate nursing students at Queen’s University Belfast. Six items of this questionnaire were comprised of Likert scale items, for example: I strongly agree, I agree, I am uncertain, I disagree, and I strongly disagree. The seventh item provided nursing students with a free-text comment box to express their overall impressions about podcasting in nurse education.

**Fig. 1:**
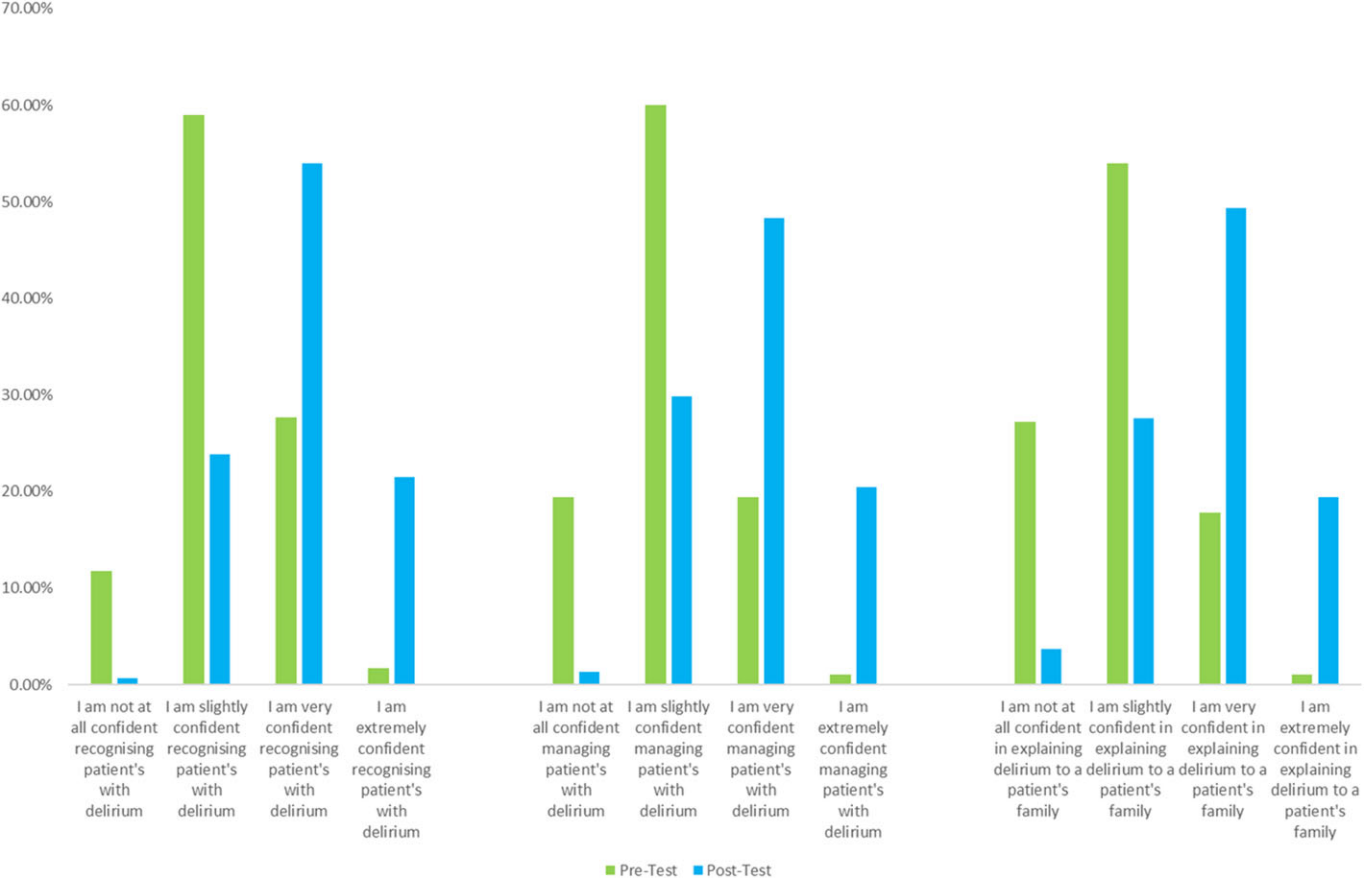
Perceived Professional Confidence about Delirium in Nursing Students Pre/Post Test (*n* = 298)

A copy of these questionnaires, and corresponding study results, can be viewed in Table 2 (delirium knowledge questionnaire), Fig. 1 (perceived professional confidence about delirium) and Table 3 (delirium podcast evaluation questionnaire).

**Table 2:** Descriptive Statistics of Delirium Knowledge Questionnaire (DKQ) in Nursing Students Pre/Post-Test (*n* = 298)

DKQ Item	Pre-Test Score (% Correct Answers)	Post-Test Score (% Correct Answers)	+/− Difference (% Correct Answers)
**A. Items related to knowledge about the presentation, symptoms, and outcomes of delirium**			
1. Fluctuation between orientation and disorientation is a typical feature of delirium **(T)**	96.64%	99.26%	+ 2.62%
2. Symptoms of depression may mimic delirium **(T)**	78.04%	93.63%	+ 15.59%
3. Patients never remember episodes of delirium **(F)**	77.70%	90.60%	+ 12.90%
4. Delirium never lasts for more than a few hours **(F)**	94.93%	96.22%	+ 1.29%
5. A patient who is lethargic and difficult to rouse does certainly not have a delirium **(F)**	88.85%	96.60%	+ 7.75%
6. Patients with delirium are always physically and/or verbally aggressive **(F)**	95.95%	97.74%	+ 1.79%
7. Patients with delirium have a higher mortality rate **(T)**	62.16%	92.08%	+ 29.92%
8. Behavioural changes in the course of the day are typical of delirium **(T)**	94.58%	96.96%	+ 2.38%
9. A patient with delirium is likely to be easily distracted and/or have difficulty following a conversation **(T)**	96.27%	98.48%	+ 2.21%
10. Patients with delirium will often experience perceptual disturbances (e.g. visual and/or auditory hallucinations) **(T)**	98.31%	99.62%	+ 1.31%
***Section A Overall Score***	**88.34%**	**96.12%**	**7.78%**
**B. Items related to knowledge about causes and risk factors of delirium**			
11. A patient admitted with pneumonia and having diabetes, visual and auditory disturbances has the same risk for delirium as a patient admitted with pneumonia without co-morbidities **(T)**	71.53%	75.67%	+ 4.14%
12. The risk for delirium increases with age **(T)**	82.71%	97.35%	+ 14.64%
13. A patient with impaired vision is at increased risk of delirium **(T)**	38.98%	82.13%	+ 43.15%
14. The greater the number of medications a patient is taking, the greater their risk of delirium **(T)**	86.39%	96.20%	+ 9.81%
15. A urinary catheter reduces the risk of delirium **(F)**	86.05%	90.49%	+ 4.44%
16. Poor nutrition increases the risk of delirium **(T)**	91.84%	98.10%	+ 6.26%
17. Dementia is an important risk factor for delirium **(T)**	94.56%	95.06%	+ 0.50%
18. Diabetes is an important risk factor for delirium **(F)**	14.97%	54.20%	+ 39.23%
19. Dehydration can be a risk factor for delirium **(T)**	96.94%	99.23%	+ 2.29%
20. Delirium is generally caused by alcohol withdrawal **(F)**	81.97%	85.06%	+ 3.09%
21. A family history of dementia predisposes a patient to delirium **(F)**	38.70%	57.85%	+ 19.15%
***Section B Overall Score***	**71.33%**	**84.67%**	**+ 13.34%**
**Items related to knowledge about delirium prevention and management strategies**			
22. Treatment of delirium always includes sedation **(F)**	91.44%	95.79%	+ 4.35%
23. Daily use of the Mini-Mental State Examination (MMSE) is the best way for diagnosing delirium **(F)**	22.26%	71.26%	+ 49.00%
24. Providing as much staff as possible to take care at the patients’ bedside is an important strategy in the prevention of delirium **(F)**	71.13%	84.23%	+ 13.10%
25. The use of physical restraints in patients at risk for delirium is the best way to ensure their safety **(F)**	95.19%	97.69%	+ 2.50%
26. Encouraging patients to (correctly) wear their visual/hearing aids is necessary to prevent delirium **(T)**	75.60%	94.62%	+ 19.02%
27. Adequate hydration is an important strategy in the prevention of delirium **(T)**	98.97%	100.00%	+ 1.03%
28. The maintenance of a normal sleep-wake cycle (e.g., avoidance of sleep interruption) is an important strategy in the prevention of delirium **(T)**	98.28%	98.84%	+ 0.56%
29. The use of haloperidol in preoperative surgical fracture patients is a way to prevent delirium **(T)**	48.97%	73.36%	+ 24.39%
30. The stimulation of patients to perform different activities at the same time is a way to prevent delirium **(F)**	35.17%	57.75%	+ 22.58%
31. Keeping instructions for patients as simple as possible is important in the prevention of delirium **(T)**	86.21%	93.41%	+ 7.20%
32. Early activation/ambulation (e.g., getting patients out of bed as soon as possible) of patients is an important strategy in the prevention of delirium **(T)**	65.17%	84.88%	+ 19.71%
33. Providing patients with familiar objects (e.g., photos, clock, newspaper) is important to prevent sensory deprivation **(T)**	98.97%	99.22%	+ 0.25%
34. Avoid eye contact in the prevention of delirium because it can be seen as a threat **(F)**	86.21%	94.96%	+ 8.75%
35. Keeping oral contact with the patient is an important strategy in the prevention of delirium **(T)**	86.55%	93.41%	+ 6.86%
***Section C Overall Score***	**75.72%**	**88.53%**	**+ 12.81%**
**Student’s Overall Average Score (*n*** **= 298)**	**77.95%** **(M = 27.28)** **(SD = 3.24)**	**89.48%** **(M = 31.32)** **(SD = 2.43)**	**+ 11.54%** **(4.04)**

**Table 3:** Descriptive Statistics of Delirium Podcast Evaluation amongst Nursing Students (*n* = 298)

Statement	Result	(N=)
I **strongly agree** that the podcaster presented material clearly	76.85%	229
I **agree** that the podcaster presented material clearly	21.81%	65
I **am uncertain** that the podcaster presented material clearly	0.67%	2
I **disagree** that the podcaster presented material clearly	0.00%	0
I **strongly disagree** that the podcaster presented material clearly	0.67%	2
**I strongly agree** that the podcast content provided was straight-forward and easy to understand	75.51%	225
**I agree** that the podcast content provided was straight-forward and easy to understand	23.15%	69
**I am uncertain** that the podcast content provided was straight-forward and easy to understand	0.67%	2
**I disagree** that the podcast content provided was straight-forward and easy to understand	0.00%	0
**I strongly disagree** that the podcast content provided was straight-forward and easy to understand	0.67%	2
**I strongly agree** that the podcast met my learning needs	76.51%	228
**I agree** that the podcast met my learning needs	19.81%	59
**I am uncertain** that the podcast met my learning needs	3.02%	9
**I disagree** that the podcast met my learning needs	0.33%	1
**I strongly disagree** that the podcast met my learning needs	0.33%	1
**I strongly agree** that I would recommend this podcast to others	76.18%	227
**I agree** that I would recommend this podcast to others	21.81%	65
**I am uncertain** that I would recommend this podcast to others	1.34%	4
**I disagree** that I would recommend this podcast to others	0.67%	2
**I strongly disagree** that I would recommend this podcast to others	0.00%	0
**I strongly agree** that the duration of the podcast was appropriate	47.32%	141
**I agree** that the duration of the podcast was appropriate	32.55%	97
**I am uncertain** that the duration of the podcast was appropriate	8.38%	25
**I disagree** that the duration of the podcast was appropriate - it was too long	11.75%	35
**I disagree** that the duration of the podcast was appropriate - it was too short	0.00%	0
**I will** listen to this podcast more than once	78.19%	233
**I will not** listen to this podcast more than once	21.81%	65

### Ethics

This study received ethical approval in May 2020 by Queen’s University Belfast, Faculty of Medicine, Health and Life Sciences Research Ethics Committee (Ref: MHLS 20_53). Participants did not provide verbal or written consent but were informed that they were under no obligation to complete any of the questionnaires. Participants gave their consent to complete the questionnaire when they actively accessed the survey web links.

### Consent/recruitment

All students (*n* = 320) received information about this research project via email by an individual independent to the study. This approach concorded with many of the asynchronous learning activities that were taking place during the COVID-19 pandemic. Students were also informed about the opportunity to participate in this pre/post-test study. It was made clear that their participation in this research was voluntary and would not affect their module grade.

Participants were advised that the pre-test should be completed immediately before downloading and listening to the podcast, while the post-test completion should ideally take place as soon after listening to the podcast as possible. Due to the nature of asynchronous remote learning, it was not possible to determine how soon before or after listening to the podcast that nursing students completed the questionnaires. While we did not collect any demographic detail from students, we did ask them to provide their student identification number at the beginning of each questionnaire. This enabled the research team to pair the data to carry out specific statistical tests.

Students did not have to sign written consent forms for this study but were informed that they were under no obligation to complete either questionnaire. It was implied that students gave their consent to complete the questionnaires when they actively accessed the web-links provided on their module homepage. Student participants were required to use their own laptop, computer tablet or mobile phone to complete the surveys. There was no option to complete paper-based questionnaires.

### Data analysis

Descriptive statistics were used to analyse overall pre-test and post-test scores for both the DKQ and perceived professional confidence questionnaire to determine the overall impact of the delirium awareness podcast. Descriptive statistics were also used for the podcast evaluation which occurred post-test. Inferential statistical analysis was undertaken to illuminate pre and post-test differences across the three core sections of the DKQ and the three statements regarding perceived professional confidence about delirium. Dependent paired t-tests were used to establish statistical significance in the DKQ and perceived professional confidence questionnaire (*P* < 0.05). In these two questionnaires, the average differences between participant’s overall scores, before and after listening to the podcast, were also calculated to highlight the extent of improvements in knowledge and confidence.

## Results

The questionnaires were completed by 298 nursing students in total, giving a response rate of 93.13%. At the time of listening, all participating students had completed two clinical placements, lasting 10 weeks in total, as part of their degree programme. All participants were enrolled as a nursing student on one of the four pathways: adult nursing, mental health nursing, children’s nursing, or learning disability nursing.

In terms of knowledge, the overall average score showed a significant increase of 11.54% (*p* < 0.001) between pre and post-tests. The standard deviation was 3.24 pre-test and 2.43 post-test. Descriptive statistics from the DKQ can be viewed in Table 2. Student perceived professional confidence about delirium was also extremely positive post-podcast and descriptive statistics from this 3-item questionnaire can be viewed in Fig. 1. Finally, students were asked to complete a short 7-item questionnaire about the use of podcasting for delirium education post-test. Descriptive statistics from this questionnaire can be found in Table 3.

### Knowledge about delirium

The presentation, symptoms, and outcomes of delirium (Section A) of the DKQ was positively answered by students scoring a mean of 88.34% in the pre-podcast test. Student knowledge surrounding; mortality rate in those with delirium (62.16%), patients never remembering episodes of delirium (77.70%) and symptoms of depression mimicking delirium (78.04%) was inferior in comparison to knowledge of other questions. These three items improved the most post-test. After listening to the delirium awareness podcast the students average score for section A increased to 96.12% overall. Knowledge improved by an average of 7.78% among student nurses after participation in the podcast (*P* = 0.007).

Students’ knowledge surrounding delirium aetiology is captured in Section B. On average, nursing students scored the least in this section pre-podcast with an average score of 71.33% overall. Questions with the weakest averages encompassed those in relation to impaired vision (38.98%), diabetes (14.97%) and those with a family history of dementia (38.70%) being linked to delirium. This items improved the most post-test. Post-podcast knowledge of aetiology increased by 13.34% achieving an average score of 84.67% (*P* = 0.003).

Student nurse’s knowledge of delirium prevention and management is studied in Section C of DKQ. Pre-podcast findings show students scored 75.72% on average. Questions answered regarding pharmacological intervention in pre-operative patients, diagnostic tools, and the stimulation of patient performance were amongst the poorest answered. Post-podcast knowledge had a vast increase in questions relating; to diagnostic tools (49.00% increase), the use of haloperidol in pre-operative fracture patients (24.39% increase), the stimulation of patients to preform activities (22.58% increase), early ambulation of patients (19.71% increase) and finally the importance of visual or hearing aids (19.02% increase). On average student scores increased by 12.81% in this section post podcast (*P* = 0.003).

Based on the average of the student’s overall score, the post-test showed improvement across all 35-items in the DKQ. The students overall average score increased by 11.54% noting an average overall post-test result of 89.48% (*P* < 0.001).

### Perceived professional confidence about delirium

Nursing students perceived professional confidence about delirium increased after listening to the podcast (Fig. 1). Overall, only 29.21% of respondents stated they felt ‘very’ or ‘extremely’ confident pre-podcast in delirium recognition and this increased to 75.71% of students’ post-podcast, representing a 46.50% increase. This was statistically significant (*P* = 0.004). Regarding, delirium management, only 20.47% students felt confident before listening to the podcast and this increased to 68.79% feeling they were ‘very’ or ‘extremely’ confident post-podcast. This represented an increase of 48.32% and this was significant (P = 0.004). The final item was around communicating with someone’s next-of kin or care partner about delirium. Student confidence in this domain improved by 50.71% overall, with only 18.08% confident pre-podcast and a 68.79% confident post-podcast. This finding was also statistically significant (*P* = 0.002).

### Podcast evaluation

Nursing students positively evaluated the use of podcast for promoting their knowledge and confidence about delirium (Table 3). Overall, 98.66% (*n* = 294) of respondents believed that the podcaster presented material on delirium clearly and that the podcast content was straight-forward and easy to understand. In total, 96.32% (*n* = 287) of nursing students believed that the podcast met their learning needs about delirium and 97.99% (*n* = 292) stated that they would recommend the resource to others. Opinion was more divided on the appropriate duration of the delirium awareness podcast with 11.75% (*n* = 35) of students believing the 60-min podcast was too long and 8.38% (*n* = 25) uncertain about whether the length was appropriate. Almost four out of five nursing students (*n* = 233) stated they would re-listen to the delirium awareness podcast after the study (78.19%).

The final question provided students with an opportunity to provide some written comments about podcasting as a learning resource. 84.23% of the sample (*n* = 251) provided a response. All responses can be viewed in supplementary file 2.

## Discussion

Audio podcasts have a key advantage of being able to present information about a topic accessible at any time or location [28, 29]. Using podcasts can also be a significant learning aid for auditory learners and have been associated with improvements in knowledge and practice amongst healthcare audiences [30–34]. Despite their growing use in the healthcare education, there has been limited practical guidance about how to effectively develop and evaluate podcasts [35, 36]. In the current study, students favourably evaluated the use of a podcast to support their learning about delirium. One area that divided opinion amongst participants was around the duration of the podcast. Less than half of the nursing students (47.32%) who took part in this study strongly agreed that the duration of 60 min was appropriate. While there are no formal guidelines on podcast duration, a recent systematic review on the use of podcasts in medical education suggested educational podcasts are normally concise and last approximately 20 minutes [36]. However, there was no evidence to suggest longer podcasts are associated with poorer outcomes. In terms of this delirium awareness podcast, it would be possible to provide three concise audio recordings (i.e. 20 min each in duration) in relation to; what is delirium, delirium recognition and delirium management as described in Table 1.

As an educational tool, the delirium awareness podcast facilitated students to develop a deeper understanding about the topic. In particular, the goal of this podcast was to facilitate student learners to actively construct their own knowledge and meaning from listening, for example applying learning from this podcast to their past personal or professional experiences in healthcare. This underpinning constructivist theory is a popular pedagogical approach which has previously been aligned to podcasting in higher education [37]. Recent literature reviews on podcasting within nursing and medical education have found only a small number of studies were underpinned by a pedagogical theory or alternative learning framework [36, 38–40].

In this study, statistically significant improvements were recorded in year one nursing student knowledge about delirium post-podcast across all three themes: knowledge about the presentation, symptoms, and outcomes of delirium, knowledge about causes and risk factors of delirium and knowledge about delirium prevention and management strategies. Most importantly, nursing student knowledge improved in all 35 items post-podcast. These findings are reflective to the findings of the research team’s original co-designed delirium awareness programme [20]. In this previous study, nursing student overall knowledge increased by 10.00% post-education and this is comparable to this current study with nursing student overall knowledge increasing by 11.54% post-podcast. In relation to perceived professional confidence about delirium, nursing student confidence about delirium significantly increased post-podcast. These findings are also consistent with the team’s original co-designed delirium awareness programme [20].

The results of this study are also consistent with previous research on delirium education with qualified healthcare professionals [16, 18, 41–44]. With consideration to the pre-registration teaching of healthcare professionals, approaches to delirium education remain inconsistent and there is a paucity of investigation about the impact on undergraduate nursing students specifically [45, 46]. Findings from a recent Delphi-study indicated some important areas for development in delirium education amongst undergraduate nursing students at Universities in Scotland [17]. Key findings from this Delphi-study included: the lack of education that differentiated between dementia and delirium, the inclusion of delirium education as a self-taught component only, the lack of specific learning outcomes related to delirium and the reliance on clinical placement areas to provide education about delirium in the absence of University-teaching about the subject [17]. These limitations in the provision of undergraduate nursing delirium education are alarming because appropriate education has the potential to improve student recognition, management, and prevention of delirium. The present study demonstrated a novel approach to undergraduate nursing delirium education through the provision of an audio podcast.

### Recommendations

The COVID-19 pandemic has changed the way in which nursing students are educated due to more limited opportunities for face-to-face teaching [47]. In response, nurse educators have had to move much of their teaching online and offer alternatives to previous methods of delivery [48, 49]. While the planning of meaningful learning can be challenging, this study has demonstrated that the provision of a co-designed educational podcast has the potential to facilitate student nurse learning about a complex medical condition. Audio podcasts also have the added benefit of reducing learning that takes place in front of a digital screen, something which has been identified as a possible risk factor for future eye disorders [50]. The study authors recommend the use of audio podcasts as a tool to deliver asynchronous learning opportunities for nursing students. With consideration to the aforementioned evidence-base, it is apparent that student nursing education about delirium is important given its high prevalence across all clinical settings and it is our recommendation that delirium education forms a mandatory part of all nursing curricula. In addition, the DKQ [26] may also offer nurse educators with a ready-made 35-item quiz to evaluate student knowledge about delirium using formative assessment.

### Strengths, limitations and future research

This study is novel and to our knowledge there have been no studies that have investigated the impact of a delirium awareness podcast amongst year one nursing students, this is a noteworthy strength. In addition, the podcast led to significant improvements in student nurse knowledge about delirium post-intervention and this has the potential to support participants in the timely recognition and management of delirium. The original delirium awareness can be accessed and shared with other cohorts of nursing via supplementary file 1. Finally, this study reports on a high number of participants (*n* = 298) at the same stage of their nurse training (year one students with 10 weeks clinical placement experience). Although this represents a large cohort, generalisability of the findings may be limited because the sample was from one university in Northern Ireland. This study also did not collect any demographic data from nursing students which could have detailed previous care experience of participants. Due to the COVID-19 pandemic, it was not possible to compare the delirium awareness podcast to a control group or alternative teaching delivery method, for example face-to-face delivery. Further research comparing an audio podcast verses an alternative method of education would strengthen the evidence-base. A recommendation for future research is comparison of delirium education interventions across multiple universities or multiple groups of healthcare professionals. It would also be useful to follow-up with nursing students in this and future studies to determine if the extent to which they retain their knowledge about delirium. Qualitative research may also be important in supporting educators to understand how student nurses apply their knowledge about delirium during their clinical placements.

## Conclusion

Low costs coupled with ease of production have made podcasting an appealing approach to pre-registration healthcare education. There have already been several studies which have demonstrated that podcasts are both feasible and acceptable to a wide variety of learners. While podcasting is clearly an encouraging approach to education little is still known about how to best develop and evaluate podcasts in healthcare education.

This study highlights how a 60-min audio podcast about delirium can enhance both knowledge and confidence about the condition. The apparent lack of supporting research on innovative educational approaches to undergraduate nursing delirium education is surprising given the high prevalence of the condition in hospitals and care homes. There continues to be an urgent need to improve delirium knowledge in healthcare education. This will support nursing students to prevent, recognise and manage delirium in their patients more effectively.

## Supplementary Information

**Additional file 1.** Download an audio file of the delirium awareness podcast that was used in this research.

**Additional file 2.** Download a copy of student open-text comments about the delirium awareness podcast.

## Data Availability

The datasets generated and/or analysed during the current study are not publicly available as per our ethical approval but are available from the corresponding author on reasonable request.

## Abbreviations

DKQ: Delirium Knowledge Questionnaire
QUB: Queen’s University Belfast
NICE: The National Institute for Health and Care Excellence
RCN: Royal College of Nursing
4AT: 4 A’s Test: A Rapid Clinical Test for Delirium
