# Supplementary material for: Evaluation of a delirium awareness podcast for undergraduate nursing students in Northern Ireland: a pre−/post-test study
Source: BMC Nurs. 2021 Jan 13;20:20. doi: 10.1186/s12912-021-00543-0 (PMC7804906; doi:10.1186/s12912-021-00543-0)
Supplement: Supplementary file 2 — Additional file 2. Download a copy of student open-text comments about the delirium awareness podcast. [file 12912_2021_543_MOESM2_ESM.docx]

**Supplementary File 2: Podcast Evaluation: Open-Text Comments**

**Please provide some comments about podcasting as a learning resource:**

- Very helpful content to explain complex information in an anecdotal/situational-like way.
- Podcasting is useful as it provides a more indepth knowledge of the subject/ topic, explaining it further.
- it is a good way to learn
- It is a brilliant resource, especially with a lively and engaging podcatser such as Gary.
- I think podcasts are a great way to get a break from reading from lecture slides ect and just listen especially during this period of COVID19
- A very interesting podcast. I had heard about delirium but knew very little about it until now. A very efective teaching tool.
- very clear and interesting informarion
- very effective as the condition is teaching can be elborated on greatly
- It is an enjoyable learning resource
- this podcast was too long
- I though the podcast was very engaging and was a good way to be able to learn as a remote learning resource.
- It was straightforward, helped my understanding and an interesting way to learn.
- clearly followed. summary document would concrete ideas in my head better.
- it was a nce way to recieve the information
- It is a good learning resource but was slightly too long couldnt listen to it in one sitting. If you are a visual learner it can be hard to process the information
- Excellent resource.
- Can be listend to at home, driving, walking. Excellent resource that can be used at times convneient to learner outside of scheduled learnign time. Highly supportive learning resource.
- Really enjoy the way Gary delivered the podcast. Very educational and interesting to listen to. Will certainly listening again to make sure I will understand all the signs, symptoms, prevention and treatments. all information necessary to educate about dementia is included in the podcast. Couldn't expect anything less from Gary. A brilliant tutor and so knowledgeable. I always enjoy his lectures and take information on board easily. thank you for sharing.
- Quite useful
- useful knowledge and kept interesting by use of personal experience
- useful if you are an audible learner
- Information was clear and easy to understand.
- It was very educating and interesting. It helped me to expand my knowledge about delrium.
- Very effective for auditory learners. May not be as effective for visual learners who require powerpoints, pictures, etc.
- I found that I was interested the entire time as it was about an actual real life event. I enjoy podcasts as it feels just like having a conversation as if we were back in the lecture hall and I find that I learn a lot from thee.
- It is a useful method for students who are auditory learners.
- it was good as you can listen to it at any time however, personally after a period of time i would disengage due to not having anything to look at and visually learn from. due to this amd it being a podcast i was able to listen bit by bit and dound it very interesting and imfomative.
- I like Gary's own examples of experiencing patients with delirium. The podcast was easy to listen to but I dont think I would listen to it all again as it is long. It is good as a learning resouce as you can pause the podcast and take down notes.
- was very clear and helpful
- It was very helpful, a good summary of information and was very intresting.
- I found it useful in explaining the information and helpful during this time as it keeps the informatiion interesting
- The podcast was different than usual but helped alot.
- Gary did a great job with the podcast, really helped me understand it better! Thank you
- very good way of learning, can do other things while still listening and picking up key points.
- Fantastic resource from Gary, as expected. easy to listen to and easy to learn from.
- This podcast was extremely interesting and easy to listen to
- interesting but the duration was too long
- It was an interesting method of teaching as it was a lot easier to follow and I was able to do other activites while listening to it eg making lunch
- i think it is a good idea to use for learning as it is enjoyable!
- Good information and easy to listen to.
- Its easy to listen to as you can listen to it anywhere
- i enjoyed the podcast as it can be adapted into many daily life activities
- very good and helpful easy to understand
- Very interesting. Very well delivered. Thank you Gary for another interesting and much needed lesson.
- I thought that listening of podcast about dellirium would be very difficult, and I didnt think it could be interesting without any interaction, just listening, especially when I saw it was 70 mins long! Gary took teaching method to the next level. I have listen all the stories with interst and it was so fascinating that I didnt even realized when this time was gone. I have learned about dellirium a lot, and I am very ready to use my knowledge in practice. Thank you Gary
- VERY USEFUL AND WOULD LISTEN AGAIN
- I feel as though they are very effective at getting the message across
- I enjoy the use of a podcast as i can listen more than once to go over things i didnt catch the first time
- very effective resource for learning and was clear to understand.
- The podcast provided very useful information and I found it very helpful when learning about delirium.
- hard to concentrate for that length of time with distractions
- I find this helps me learn better as it is personal stories and I find I can remember things or relate better when it is like this
- WAS VERY USEFUL AS CAN USE IN CURRENT EMPLOYMENT.
- clear very educational material, used personal experience easy to remember
- Good relevant information provided
- IT WAS GOOD AND A WELCOMED STYLE OF LEARING APOSED TO THE POWERPOINTS AND VIDEOS
- very interesting way of learning compared to powerpoints!
- It is easy to listen to and you can listen to it while doing anything.
- Very informative and easy to understand
- I really like this form of learning and find it very useful being able to pause and rewind information when trying to learn. I hope podcasts become a regular thing, thankyou!
- very useful and provided good information
- Excellent podcast Gary, as always easy to understand and relatable content. Perfect for giving family members an explanation also. Thanks for taking the time to put together. Look forward to others like it
- Easier to focus on a podcast than written notes
- podcasting is good as you can do it anywhere
- This was a thought provoking and engaging podcast. The personal material and stories made it very relatable. It is frightening to think how easily it could be misdiagnosed as dementia, especially for those affected who have no one to advocate for them.
- Clear information Easy to recognise the signs of delerium Personal story was effective explaination
- Jenna It was an efficient way to learn
- This was a great experience to learn delirium
- i found it very useful
- i really enjoyed this
- Very useful to understand how complex the issue of delirium in patients is
- I found it an extremely beneficial learning tool
- very good for auditory learners, very benificial
- Would like to hear more of them. Or to have some as reinforcement after completing a lecture. Enables learning to become extremely versatile can listen exerciseing/ cooking or even putting away Landry.
- Although the podcast was interesting and met my learning needs, I found that the podcast could've been slightly shorter as I had to take breaks throughout to ensure concentration.
- I liked listening to the podcast. I felt I learned more by listening to a familiar voice than reading powerpoints.
- I enjoy listening to podcasts and it is an interesting way to learn as you can do things while listening or make notes
- I found that the podcast was an excellent learning experiencing. I thought I might find it hard to concentrate due to the lack of visual engagement but this was not the case. The length of the podcast ws appropraote for the in depth information that was provided. I found that the pace and tone was just right for the infromation and I found it useful that I could pause it if I needed to take down extra notes. Overall Gary done an excellent job in delivering this podcast.
- i found that the podcast was an interesting and much more interactive way of learning the content as i was able to pause the podcast at any point to make notes and i can also listen to the podcast again to refresh my memory or to listen too again if i am unsure of anything. I also found that it was easy to listen too and the information was easy to understand.
- great way to make learning more interesting
- Enjoyed the podcast, it was explained clearly.
- it was very informative clear and consise, easy to retain and provided futher skills that will contribute to my future career in nursing
- Anna i loved this method of learning. with having a baby at home, it allowed me to listen while continuing to meet my childs needs. i did not have to sit down and follow along a power point.
- It was very long
- Very informative especially when its told from someone with a personal experience.
- A straightforward and simple way of teaching, made learning easier.
- The podcast covered the topic well. - It was clear and easy to understand, it wasn't too heavy. Gary tells personal stories and jokes which makes it easy to engage and stay focused. - I now feel more confident on the topic of delirium.
- Thanks Gary
- Great way of learning, easily managed and can listen at own leisure. Great input from Gary
- its a good way of getting materials across
- Great podcast. Thouroughly explains info with anecdotal evidence which makes it easy to understand.
- The Podcast was an enjoyable alternative to slides and the added bonus of being able to access it without having to stay in one place was a welcome difference. overall an enjoyable learning experience.
- it was good as the stories and personal experiences helped conect the information to clinical setting
- I enjoy being able to listen to podcasts while doing other things around the house. Helps me take it in rather sitting in one place not being able to decide when to listen or take a break
- This podcast was very informative and the content was very clear and easy to understand.
- i think it is an excellent tool as it allows us to stop and start to make our own notes along the way. also, if you misheard something you can skip back
- Good way of learning
- Gary explained the topic with great enthusiasm and used terminology which was easy to understand. The examples of his clinical and personal experience with dementia made it easy to listen to and enjoyable despite it being a distressing and sad topic. Thank you
- very helpful
- It is a useful resource as it means you can drop in when you wish and break it down into more manageable chunks. It would have been better as a video with images to look at to stay stimulated just due to the length of it.
- very good
- I find it hard to concentrate while listening to podcasts. I much rather have visual images alongside audio
- Was a brilliant podcast to listen to.
- I enjoyed this podcast!
- I found it very helpful as i am more of an audio learner so stories and listening to people helps me to learn
- really enjoyed the stories the pace it was read at and repeating of facts
- Very good and informtaive
- SIMPLE, STRAIGHT TO THE POINT, EFFECTIVE! PERSONALLY I WOULD NEED TO LISTEN MORE THAN ONCE BUT THAT'S NOT UNUSUAL FOR ME.
- This took me a while to complete as I kept getting distracted perhaps it was the duration of this particular podcast
- Very useful learning tool as I was unaware of recognising delerium before, very straightforward and interesting teaching and easy to listen to.
- great podcast! very informative, i was ab le to make notes while listening, the video
- recordings throughout and storys helped me engage better, compared to just following a powerpoint. i learnt alot thank you!
- Easy to learn from, practicaal for people who are on the move a lot as it can be listened to anywhere. Practical for parents as it can be paused if needs be and can come back to it when able.
- Incoportating personal stories into the podcast, makes it easier to learn in my opinion as you can relate facts to real life.
- I found this podcast to be very helpful, and more benefiticial to hear someone explain a topic rather than to read notes.
- I liked that it gave mutiple examples. I thought I knew a bit about delirium but I learned loads from this.
- Very useful as you can listen to it on the go and can replay to listen to information again.
- The podcast was absolutely brilliant and I really enjoyed listening to it, even if it lasted over an hour! I know so much more about delirium now!
- i Found the podcast to be extremely useful as I can go back to points where I missed. Podcaster explained delirium very well and was overall a very interesting podcast.
- Really good, informative and interesting podcast
- thanks
- This podcast was very informative and points made were easy to understand with sentences not made complicated, making it enjoyable to learn from. The use of a story really made it interesting and was very touching. It was easy to follow and listen too. The topic was fascinating and I have defitely been taught information i never knew before to build on my knowledge. I would highly recommend this podcast to others and will definitely be listening to it many more times.
- it was great, i was able to stop ans start it podcast and tupe the notes that i need
- very touching and personal
- Very helpful. Podcastor related to their own experiances which make it easier to understand.
- I thought it was amazing, it covered everything I needed to know about Deliurm and it gave me a better understanding of it also. The use of examples where really good in developing my understandment of Deliurm. The language of the podcast was appropriate and I was able to understand it clearly. I thought it was interesting and very well delivered.
- A good learning resource due to COVID19 circumstances.
- i thought it was incredibly effective. it held my attention, i was able to pause and rewind when i needed to (homeschooling! I felt it was probably more effective for me as a learning technique than lectures as it was so well explained, i had extra time to take notes and listen again, and there were no distractions causing me to miss some content
- the resource was easy to listen to. it was easy to follow and was elpful with learning because its easier to learn while listening to someone. the podcast was very well explained.
- 10/10 would recommend as a learning resource go gary
- I thought that the podcast was beneficial as it went into detail about what delirum is, how to spot, manage and prevent it and aslo inculded personal experinces.
- I found it very helpful and easy to follow. It is great as I can follow at my own pace while making notes and I can listen again in case of any confusion. Overall a very helpful way of grasping a topic.
- Under the current circumstances, it was nice to receive the material in a varied way. However, it would be good if it was accompanied by some written material too as it was hard to keep stopping and starting the podcast to take notes.
- effective way to provide information, however can be jard to keep focused on it
- VERY EASY TO UNDERSTAND
- i think this is a great way of learning nursing content. i love how simple the information was relayey.
- The podcast was engaging and the information delivered was well presented and easily followed. Overall the podcast was very interesting and met my learning needs.
- The podcast was very engaging and it was nicely laid out with clips of other speakers to aid learning and understanding of delirium
- A good explanation of delirium enjoyed the use of personal experiences to give an example of delirium. very easy to listen to and take in information.
- I usually listen to the radio while cleaning, but I listened to this podacast instead. My mother listened as well, and we both really enjoyed it as it was very interesting and it was a really easy way of learning at home!
- Tone of voice was very good, very informative in such a was that it was easy to follow and understand, as well as allowing me to develop and understanding. As a Dyslexic student this was very useful to listen to and helped to stay foucued, especailly during the circumstances at the moment, while I was also making notes- a very good way of learning for me. Thank you.
- Tone of voice was very good, helpful to understand and I was able follow each step to develop understanding , as a dyslexic student it was very good an inforative to hear, as well as listening to the Pod Cast during the current sutuations made it easier to pay attention and complete work. Thank you.
- Very useful as a learning resource. Multiple resources can be added. Can be paused and restarted allowing for note taking. A very personal experience - put earphones in and just listen. No distraction from images etc
- Niamh very good intresting podcast a bit too long and drawn out but very well told
- really interesting, great quiz helpful with revsion!
- Gary always makes his lecture easy to follow, fun and very informative.
- it was oksy but not the best learning tool as took too long and lost intrest
- I think podcasting is a good learning resource for many people, however, for me I learn better by seeing information visually as well as listening. If I am just listening to information I struggle to concetrate and take in the relevant information.
- i like this method of learning information. Story telling helps me to remember information so the personal stories helped alot. The podcast was spoken in a way it was easy to understand and make brief notes as it went along.
- real life experiences helped me understnd how this can be adapted in practise.
- intresting podcast but was very long
- It was an interesting and different way to learn. I enjoyed the podcast and the quiz, I feel like the quiz consolidated my knowledge.
- this podcast was very interesting and engaging
- this podcast was very interetsing and engaging
- Very benefical as it was informative and interesting.
- very useful in relation to nursing good that we were able to listen to it numerous times to get a better understanding on certain topics
- Overall podcasts are very helpful for me. In relation to this one, I thought this was a brilliant podcast and brought so much more light to the issue. From previsouly having family members experience delirium it was never communicated to us as a family and many of the staff seemed to brush it off. It has given me such a greater awareness of delirium and how I would approach it differently if I experienced it in practice.
- Very informative
- great
- It was good as i was able to listen to it while getting other tasks done but it was very long to sit there and listen too.
- Podcast was a good idea and it was nice to have a different method for learning material to be delivered. The podcast also meant that you were able to do other things while listening.
- i enjoyed using it and info was appropriate. an accompaning summary written down of the podcast woud definitely help me understand the topic fully.
- This was a great podcast, it was very informative and relevant to the chosen topic however I feel it could have been condensed slightly as I found it hard to concentrate once I listened past 45mins. It may also be a greater help to us if there were a few ‘take-home message’ bullet points written for us to view under the podcast. But i did enjoy it, Great job Gary!
- very clear and witg the use of examples made it easier to understand
- Brilliant commentary. Your enthusiasm really helped me with paying attention. I really enjoyed the podcast layout... Meant I could go about my daily chores while listening to it instead of sitting at a computer. Thank you! You should really get into narrating audiobooks.
- was easy to follow and easy to listen to. very useful tool as can listen to at any time etc.
- Was easy to listen to, great that you can re-listen and rewind if needed
- I found the podcast very informative and easy to understand.
- It was really useful and really nice to hear examples of patients' suffering from delirium from a personal experience. Even nicer though to hear how easy it can be to improve the condition. I just felt the pod cast was slightly too long.
- Very Interesting and easy way to learn however, quite time consuming as podcast was rather long and notes had to be take to remember so often many pauses and plays.
- The podcast was informative, enjoyable and met my learning requirements in relation to gaining an understanding of delirium.
- I worked with patients with delirium before, but it wasnt explained to me how it occurs and how to provide treatment or trying to avoid this situation. the podcast explained everything I wasnt aware of.
- Explained things well and the use of personal anecdotes helped connect learning to real life.
- I found the podcast to be very informative without being overloaded. I would recommend this to other students
- Enjoyed this way of learning, it was effective as you can listen to it more than once and go back to bits that are unclear to you. Also effective as you can listen while doing day-to-day activities.
- gives you the opportunity to listen to it again and again with good explanation and it hits home with examples which are so relevant
- informative and easy to follow.
- really interesting, enjoyed thos different still of learning. I feel it was very effective
- loved the podcast! its a shame i couldnt listen to it with my phone locked though as it would have been nice to listen to while out for my walk!
- I loved it!! Makes learning more interesting and hearing a person story puts it into perspective. Its hard reading so much notes all the time so this was a great break and you learn more. I tend to pause it and make appropriate notes.
- Gary as always held my attention throughout and the use of his own personal experience with his grandfather helped me understand delirium and how it can affect as well as impact an individual. Very informatative and easy to listen to.
- i find it very useful to mentally picture what's being said. I feel i need to concentrate that little harder which keeps me focused and engaged and so i take more in.
- good resource, just a bit too long.
- This was an excellent podcast, that was easy to follow and a great guidence on delirium.
- i really liked this podcast, it was nice to have a different method of learning. i enjoyed all the little stories in between it made it easy to understand. however i think it was a little long for a tutorial as the 2 quiz's with it took more time than normal in queens tutorials.
- I thoroughly enjoyed the podcast as I am both personally and professionally interested in delirium and also dementia. Gary made all information easy to understand and follow. I felt intrigued and engaged throughout and will definitely listen to it again.
- I found it really helpful and enjoyed engaging with it as a learning resource alongside participating in the pre and post podcast quizzes; as I feel that this has maximised my learning.
- I found this poscast very good as a learning resourse as it gave me the opportunity to listen to it at my own speed to enable me to take notes. As always with Gary's lectures it was enjoyable and intresting.
- I thought it was an excellent learning resource. It was easy to interact with the podcast as it was very relatable and relevant. Great learning experience!
- it was a very detailed and easy to follow podcast and i felt i learnt a lot
- The use of personal stories and the fact that Gary talked so compassionate about
- the subject made the podcast more interesting and made you want to listen on
- Very detailed, but explained in an easy way to understand
- I enjoyed this method of learning. It is helpful as we can continue to listen to a podcast more than once and listen to it while doing things such as driving or excercising. It is easy to go back to certain parts of a podcast if you are unclear about something and listen to it as many times as needed or wanted.
- I think it is a brilliant learning resource which should be valued and used more often. Everyone nowadays has earphones in when out walking or travelling hence the perfect time to stick on a podcast and absorb the content. Really enjoyed this.
- very very beneficial, loads of straight forward information was given and it was nice of gary to share a personal experience
- It was interesting especially to listen to different stories from different peoples points of view. I like podcasting as a learning resource as its easy to take down some notes while listening, and you can always pause and play it to take breaks or to listen to a point again.
- I found this extremely helpful/useful as I was able to play/pause it whenever i needed to or rewind to listen again to parts i needed to get my head round further. I was able to make notes during this podcast and everything was explained very well and in detail. Thank you! :)
- it was informative but i would if needed to hear more examoles
- easy to follow and listen to, provided a wide range of information all relevent and interesting. would reccomend.
- Personally i find podcasts interesting and benifical. However, having shorter and more direct podcasts may be more appealing. Making it easier to listen to and having the listeners full attention for the duration of the podcast.
- It's more beneficial for me than reading a PowerPoint, it's more interesting and I retain more information
- I found this podcast a great way to explain the symptoms of delerium - making it easy to understand and relate to patients I have come in contact with in work and placement.
- helpful as a learning exercise
- I found it easy to follow and it was clear to understand
- enjoyed it
- i really enjoy listening to this, i found it very usful and taught me alot of this subject which i feel was very relvent.
- The podcast was very informative, I was unaware of the symptoms of delirium, however after listening to the podcast, i feel that i would be better informed to recognise the symptoms and the risks associated. Although the podcast was long, the use of personal experiences throughout made it extremely interesting. very well done, thank you.
- It was a good learning resource, especially when there was an example given that helped me understand it better. Had to take a couple of breaks throughout the podcast as it was long, but overall it was easy to understand.
- it was new, interesting, easy to understand, and beneficial
- I thought it was a good way of learning
- I enjoyed istening to the podcast and writing down relevant notes where possible. This inabled me to increase my undertsanding of Dilierium and I have gained more confidence in how to recognise and treat it.
- Very useful and straightforward to understand
- A nice change, a break from reading
- brilliant idea for those that learn differently or have had enough of power points.
- I found the podcast very beneficial. The personal stories helped paint a clearer picture for me and allowed me to understand the signs of delirium better. I find hearing a voice explaining tutorials and lectures makes it more engaging and easier to take in the information and understand it. I would rewatch this podcast as revision and would refer to it when I am looking a better understanding of this topic.
- I find that podcasts are very helpful as you can listen to them at your own time and can go back and forth if you want to listen to certain points again. I find podcasts are very interesting and I find they are easier to gain information and to remember them as they are explained in more detail and easier to understand in my opinion.
- It was good to follow along to and take notes as you could pause it, write it down so you can see it and understand it better.
- Currently experiencing delirium with my own grandmother. From my own personal experience watching a loved one who has delirium is really difficult and can be very tough on the person's family. Listening to the podcast based on Delirium has taught me many more things that myself and my family were not told about in the ward she was previously in. The hospital that my granny was in, did not explain to my family what delirium was, what causes it and what prevents it. Therefore as a family we just had to educate ourselves by using the internet. Overall, I enjoyed listening to the podcast and feel that I could use the relevant information at home with my granny, and on placement also. Thanks for this Gary.
- Overall the podcast was very helpful. The range of personal stories and interviews held my attention and enabled the information to be more memorable and applicaple. Having bullet point lists within the podcast to give signifiacant, boileddown information was also helpful in learning the fundementals of delirium.
- Thoroughly enjoyed this podcast.
- very informative and engaging throughtout
- Podcast was very good. Gary always explains things very well with a good mix of theory, stories and metaphors.
- It was very educating and helped me to understand Delirium
- A very informative podcast Gary. The personal stories will stick with me and help me to recognise the signs and symptoms of delirium in patients. The statistics are quite shocking and it suprises me that we dont hear more about delirium. I have found it really interesting.
- Good learning resource but very long
- Very well presented. Excellent narrative.
- Information was clear and well set out, easy to understand. This podcast definitely taught me a lot.
- Very good learning resource, easy to listen to on the go. Able to listen to the podcast whenever or wherever
- I found it an interesting and enganging format for learning
- It was very informative and kept interest throughout
- This was a very informative piece of learning. Made better with being able to relate to a real life scenario from Gary's experience. Clear and concise and initially I felt 75 minutes seemed a bit long, but I honestly didn't realise the length of time had passed because it kept me engaged. Just annoyed because I accidentally hit the wrong answer in this DELQ2 about the co morbidity question!! Other than that, I will endevour to listen to this podcast again if i ever get through the mountainess canvas "to do" list that sits before me !
- I found it very engaging and helpful. You could pause, reflect and even research topics further. Very enjoyable!
- Thoroughly enjoyed this podcast it was very easy to listen to.It is clear how passionate Gary is about delirium especially from hearing about his past experiences with his Grandfather.A very serious topic but i did get a laugh at the story about the middle finger. After listening to this podcast i definitely feel more confident in recognising the signs and symptoms of delirium. Thanks Gary
- Found it very interesting and easy to listen to.
- very easy to follow, had good music, reflections from diffrent people was interesting.
- This is an excellent learning resource. When the lecture content is applied using a scensrio (or via an interactive activity), it is easier to understand and you retain more information.
- Thank you Gary.
- very useful, easy to access and use while fitting into a busy day
- really informative. important learning
- It was a brilliant podcast with super clear and effective material. However, I did feel it was slightly long and found it difficult to pay 100% attention towards the end. Maybe if it was the same length but split into 3/4 different listening resources where you can stop and pick up where you left off to maintain effective listening.
- Thoroughly enjoyed, it was worthwhile listening to this podcost. Very easy to connect with people when they provide background stories of their experiences and journeys in their nursing career.
- Really enjoy garys story telling was a great way for me to understang it,
- no powerpoint only issue
- Explained all material in a clear way that was easy to understand.
- Podcast was very useful, I made notes during it.
- Brilliant and Engaging
- very good learning source, the different stories captured my attention, straight forward and easy to understand, well delivered, so much so that my husband learnt from it too
